# Foreword

**DOI:** 10.1155/MBD.1994.i

**Published:** 1994

**Authors:** Henryk Kozlowski


### FOREWORD

The European Science Foundation (ESF) is a major international funding organisation supporting the collaboration and the meetings of European scientists. It is based in Strasbourg and it is financed by National Science Foundations of major West European countries. The programme Chemistry of Metals in Biological Systems has sponsored the Workshop on "Impact of Metal Ions on Drugs, Chemotherapeutics and Related Compounds". Almost all the lectures prersented during this meeting are published in this issue.

The discovery of the anti-tumour activity of platinum complexes promoted enormous interest in the research on many other inorganic compounds which might make good new anti-cancer agents. Cisplatinum was not the first metal complex tried out in cancer therapy. First systematic studies on the structure-activity relationship of tumour-inhibiting metal compounds and toxicity were started more than 50 years ago by Collier and Krauss. Platinum complexes, however, were found to be very efficient and became widely used in the clinic. Platinum compounds, however, are extremely toxic and in fact, they are really effective in a very limited number of cancers (testicular carcinoma and a few others). This promotes a very intensive research in the field. One approach is to improve properties of the platinum drugs (lower toxicity, higher specificity to cancer cells and more broad spectrum of cured tumours).

The new developments of platinum anti-cancer drugs, however, may not lead to new mechanisms of action and new structure-activity relationships, which are needed when new families of drugs are searched. Thus, inorganic chemists try to develop both platinum-type drugs as well as new kind of compounds (e.g. Ru or Sn-based drugs) which may act differently than cisplatin.

The field of inorganic drugs is, of course, not limited only to spectacular achievements of cisplatin. Gold compounds used for 7000 years in curing of various diseases, the question of metal ion toxicity (iron overload, heavy metal toxicity) and chelating agents, the transport of inorganic species through a living system, the effect of metal ions on existing organic drugs or the use of inorganic complexes in diagnosis (lanthanides in NMR imaging) are also important elements of bioinorganic chemistry and pharmacology.

The interdisciplinary character of this field brings together chemists, pharmacologists, biochemists, physicians, biophysicists. This broad approach to inorganic drugs gives reasearchers, especially young scientists, a great oportunity to develop new ideas and to perceive studied objects in a modern and universal way.

Henryk Kozlowski

